# Modifiers of notch transcriptional activity identified by genome-wide RNAi

**DOI:** 10.1186/1471-213X-10-107

**Published:** 2010-10-19

**Authors:** Philippos Mourikis, Robert J Lake, Christopher B Firnhaber, Brian S DeDecker

**Affiliations:** 1 Department of Molecular, Cellular and Developmental Biology, University of Colorado, Boulder, CO 80309, USA; 2 Stem Cells & Development, Department of Developmental Biology, Pasteur Institute, CNRS URA 2578, Paris, France; 3 Epigenetics and Progenitor Cells Keystone Program, Fox Chase Cancer Center, Philadelphia, PA 19111, USA

## Abstract

**Background:**

The Notch signaling pathway regulates a diverse array of developmental processes, and aberrant Notch signaling can lead to diseases, including cancer. To obtain a more comprehensive understanding of the genetic network that integrates into Notch signaling, we performed a genome-wide RNAi screen in *Drosophila *cell culture to identify genes that modify Notch-dependent transcription.

**Results:**

Employing complementary data analyses, we found 399 putative modifiers: 189 promoting and 210 antagonizing Notch activated transcription. These modifiers included several known Notch interactors, validating the robustness of the assay. Many novel modifiers were also identified, covering a range of cellular localizations from the extracellular matrix to the nucleus, as well as a large number of proteins with unknown function. Chromatin-modifying proteins represent a major class of genes identified, including histone deacetylase and demethylase complex components and other chromatin modifying, remodeling and replacement factors. A protein-protein interaction map of the Notch-dependent transcription modifiers revealed that a large number of the identified proteins interact physically with these core chromatin components.

**Conclusions:**

The genome-wide RNAi screen identified many genes that can modulate Notch transcriptional output. A protein interaction map of the identified genes highlighted a network of chromatin-modifying enzymes and remodelers that regulate Notch transcription. Our results open new avenues to explore the mechanisms of Notch signal regulation and the integration of this pathway into diverse cellular processes.

## Background

The Notch (N) cell-surface receptor is the central element of one of the handful of signaling pathways that are evolutionary conserved throughout metazoans. Notch signaling directs the development of multicellular organisms through membrane-anchored interactions between adjacent cells. The response to Notch signals varies greatly and can result in diverse biological consequences, such as cell proliferation, differentiation or apoptosis. Notch signaling is initiated by the binding of the transmembrane Notch receptor with one of its ligands, Delta or Serrate, expressed on the surface of a neighboring cell [1]. The receptor-ligand interaction induces a series of proteolytic events that releases the Notch intracellular domain (Nicd), which then translocates to the nucleus and complexes with transcription factors and co-activators to regulate target gene expression. Nicd, together with Suppressor of Hairless [Su(H)], a DNA binding protein in the CSL (CBF1/Su(H)/Lag2) family, and mastermind (mam) proteins form the core transcriptional complex of the signaling pathway, with the *Enhancer of Split *[*E(spl)*] locus being the most thoroughly characterized downstream transcriptional target. The Notch signaling pathway is modulated at many levels: Notch protein abundance, trafficking, and post-translational processing, as well as the regulated formation of repressive and promoting complexes on the DNA. The final cell fate outcome is a complex interplay between all the cellular factors that regulate Notch activity.

We designed a genome-wide RNA interference (RNAi) screen using a *Drosophila *cell culture-based system aimed to identify novel proteins that directly influence the signaling capacity of the core Notch pathway. This genome-wide RNAi screen was performed on *Drosophila *Kc167 cell cultures that were transfected with a construct that expresses a constitutively active, membrane-tethered form of the Notch receptor, NΔecn [2]. Notch pathway activity was monitored by measuring the transcriptional response of a luciferase-reporter gene cassette (*m3-luc*) containing the native promoter element of the *E(spl)m3 *gene [3], the most Notch responsive *E(spl) *target in cell culture [4].

The results of our study reveal the identity of proteins that influence the signaling output of the core Notch pathway. Employing complementary data analyses, we found 399 putative modifiers - 189 promoting and 210 antagonizing Notch signaling. These included several known Notch interactors, validating the robustness of the assay and our experimental approach. Molecules residing in the extracellular matrix (2%), the plasma membrane (3%), the cytosol (16%), and the nucleus (26%), as well as a large number of proteins with unknown function and localization (53%), were also recovered (Table 1).

**Table 1:** Cellular distribution of Notch modifiers selected by the complimentary analysis methods.

Analysis Method	Total	Extracellular	Membrane	Cytosolic	Nuclear	Unknown
NΔecn > m3-luc/con-luc (Activators)	153	8 (5.2%)	2 (2.6%)	28 (18.3%)	40 (26.1%)	73 (47.7%)
NΔecn > m3-luc/con-luc (Repressors)	130	0	5 (3.8%)	17 (13.1%)	27 (20.8%)	81 (62.3%)
NΔecn > m3-luc/m3-luc (Activators)	75	0	1 (1.3%)	12 (16.0%)	28 (37.3%)	34 (45.3%)
NΔecn > m3-luc/m3-luc (Repressors)	74	0	2 (2.7%)	13 (17.6%)	5 (20.3%)	44 (59.5%)
						
Total (discounting duplicates)	399	2%	3%	16%	26%	53%

The majority of the proteins are of unknown cellular localization and/or function. Of the annotated proteins, most are predicted to reside in the nucleus, followed by cytosolic proteins with a small percentage found in the plasma membrane and extracellular matrix.

To further analyze and categorize our dataset, the Notch signaling modifiers identified in the study were combined with physical interaction data from public databases. The interaction map that was generated revealed classes of interacting Notch modifiers such as mRNA processing and ribosomal proteins. The network analysis also highlighted a central core of chromatin regulating genes, including chromatin modifying enzymes and remodelers that interact directly with the Su(H) DNA binding complex.

## Results and Discussion

### Development of a robust assay to measure changes in Notch transcriptional activity

A reporter assay was developed to measure Notch activity in a high-throughput *Drosophila *cell-based approach. The assay consists of three components: 1) a Notch activity reporter construct with two, tandem copies of the *E(spl)m3 *promoter positioned upstream of the firefly luciferase gene (*m3-luc*) [3]; 2) the constitutively active, membrane-tethered form of the Notch receptor with the extracellular domain removed (NΔecn), driven by the viral OpIE2 promoter; 3) a control construct that constitutively expresses firefly luciferase, also driven by the viral OpIE2 promoter (*con-luc*). *Con-luc *was used to normalize signal intensity relative to transfection efficiency, cell density and viability, and general effects on OpIE2-mediated transcription.

**Figure 1:**
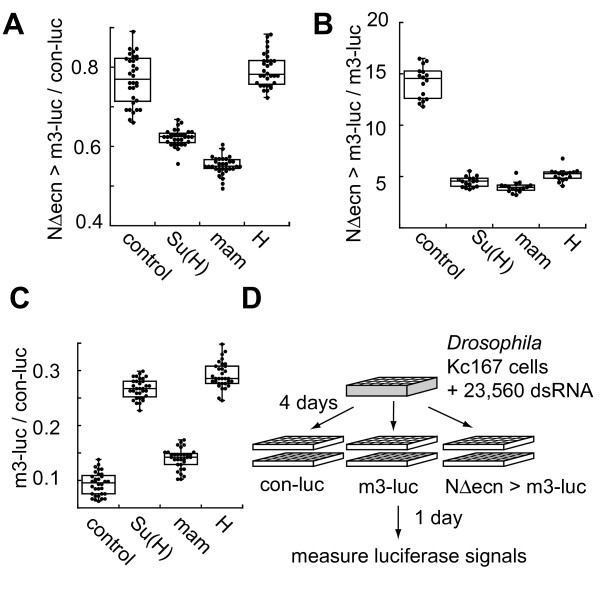
**Validation of the Notch activity reporter and application for high throughput RNAi**. Validation of the *m3-luc *reporter by RNAi targeting of known Notch pathway components. Notch induced *E(spl)m3 *signal normalized against **A**. the control viral promoter or **B**. uninduced *E(spl)m3 *promoter transcription. **C**. Uninduced *E(spl)m3 *promoter expression normalized by control promoter. For each dsRNA, 32 independent wells were measured in a 384-well plate format. Each box encloses 50% of the data with the median value displayed. The error bars mark the full range excluding the shown outliers. **D**. Schematic of the automated high throughput screen in 384-well plates. *Drosophila *Kc167 cells were incubated with a unique dsRNA per well. After a four day incubation, the cells were split into three different transfection mixes in duplicate. Firefly luciferase signals were read 24 h after transfection.

To test the sensitivity and specificity of the Notch activity assay, a series of experiments were performed in cells treated with interfering RNA targeting known components of the Notch signaling pathway. Cells were incubated with dsRNA against *mastermind *(*mam*), *Hairless *(*H*), and the major downstream co-transcription factor *Suppressor of Hairless *(*Su(H)) *and then split and transfected for three assays. N-induced (NΔecn >*m3-luc*) luciferase expression levels were measured relative to either *con-luc *(Figure 1A) or uninduced *E(spl)m3 *promoter (*m3-luc*) (Figure 1B). Uninduced promoter levels were also tested by normalizing *m3-luc *measurements with corresponding *con-luc *signals (Figure 1C).

As predicted, we found that targeting *Su(H) *and *mam *with RNAi in cells expressing activated Notch resulted in a sharp reduction of the reporter luciferase activity (Figure 1A and 1B). Conversely, knock-down of *Su(H) *increased the basal activity of the *m3-luc *reporter in the absence of NΔecn (Figure 1C). These opposing effects of *Su(H) *RNAi on *E(spl)m3 *expression are consistent with the dual roles of Su(H) as a transcriptional repressor in the absence of Notch activation, as well as a transcriptional activator when complexed with processed Nicd in the nucleus [4]. In contrast, RNAi against *Hairless *resulted in a marked decrease in the ratio of induced:uninduced signal of *m3-luc *(Figure 1B). This decrease is expected due to the specific de-repression of the uninduced Notch target promoter when *H *is knocked down (Figure 1C) and shows that there is robust Su(H)/H complex repressor activity in the uninduced Kc167 cells. The different ratios for *H *RNAi treatment obtained by the two different normalization methods (Figures 1A and 1B) highlights the additional mechanistic information that can be deduced when normalizing by the uninduced *E(spl)m3 *promoter activity. Hairless acts as a repressor in the uninduced cells, but has no apparent role in Notch activated cells.

Splitting the cells into three different assays also allows the uninduced Notch target promoter measurement to be used as an alternative and specific control for Notch induced activity. This additional control flags dsRNA treatments that may specifically affect transcription of the viral OpIE2 promoter. RNAi treatment may modulate either the signal of interest and/or the control signal and the resulting ratios may be altered indistinguishably between these possibilities. Whereas this second control will sort a subset of these dsRNAs as definitively altering Notch target transcription (positive by both normalization methods).

The Notch activity assay responded in a predictable and specific manner to RNAi of known Notch signaling components, and these data establish our experimental set-up (robotic cell transfer, normalization methods, incubation time) as optimal for detecting changes in Notch transcriptional activities.

### Genome-wide RNAi screen and data analysis

The RNAi screen was performed using a dsRNA library from the *Drosophila *RNAi Screening Center (DRSC), containing a total of 23,560 dsRNAs, targeting known and predicted gene products. After four days of RNAi treatment, cells were uniformly dispensed by robotic liquid handling into microplates containing the different transfection mixes (*con-luc, m3-luc *and NΔecn >*m3-luc*). Each assay was performed in duplicate, and firefly luciferase activity was measured 24 h after transfection.

**Figure 2:**
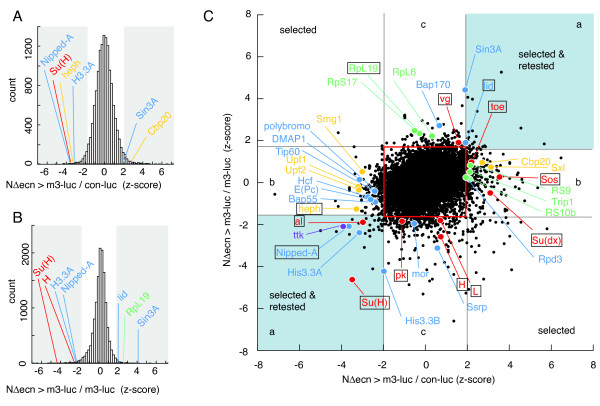
**RNAi data analysis overview**. Histograms of targeted genes binned by standard deviations from the mean (z-scores). **A**. Histogram of z-scores for Notch-induced *E(spl)m3 *reporter (NΔecn >*m3-luc*) normalized by the signal from the control reporter (*con-luc*) (Additional file 1). **B**. Signals of Notch-induced *E(spl)m3 *reporter (NΔecn >*m3-luc*) normalized by the signal from the non-induced *E(spl)m3 *reporter (Additional file 2). Cutoffs for genes selected are highlighted in grey for both A and B. **C**. Plot of the z-scores from histogram 2A on the x-axis and histogram 2B on the y-axis. Regions outside the red box are listed as potential hits and the overlaps between the two normalization methods are shaded in blue (area a). Area a. represents the subset of genes that either affect Notch induced transcription specifically or have opposing effects on induced and non-induced reporter transcription (*e.g*. Su(H)). RNAi for this overlapping set was redesigned and retested (Additional file 5). Area b. represents genes that affect both Notch induced and non-induced transcription by similar percent amounts (*e.g*. heph and Bap55). Area c. represents genes that primarily affect non-induced reporter transcription specifically (*e.g*. H, RpL19 and Bap170). Red and/or boxed genes have known genetic interactions with *Notch*. Blue are chromatin components, Yellow are mRNA processing factors, and Green are ribosomal components (*Minute *class).

For data analysis, we eliminated all wells containing dsRNA with more than one off-target, as predicted by the *Drosophila *RNAi Screening Center (DRSC). Of the dsRNA in the final hit lists, 12% contained a single possible predicted off-target and are noted in the data tables. Data from the screen were analyzed by the two complementary methods described above (see Figures 1A and 1B). Prospective hits were selected as dsRNAs that significantly perturbed the Notch induced signal (NΔecn >*m3*-luc), normalized by the control promoter (*con-luc*), resulting in 153 hits with significantly low and 130 with significantly high signals respectively (Figure 2A and Additional file 1). A complementary set of hits were selected with signals from Notch induced reporter (NΔecn >*m3-luc*), normalized by the uninduced promoter (*m3-luc*), resulting in 74 hits with significantly low and 75 hits with significantly high signals (Figure 2B and Additional file 2). Analyzing the data by these two methods provided a full spectrum of Notch signaling effectors that could be further categorized by their respective activities. Hits that scored in both normalization methods represent the subset of genes that either affect Notch induced transcription specifically or have opposing effects between induced and uninduced transcription, such as *Su(H) *(Figure 2C, area a.). Hits that scored only for Notch induced signal (NΔecn >*m3-luc*) normalized by the viral promoter (*con-luc*) primarily selected for genes that affect the Notch induced and uninduced transcription by the same percentage (Figure 2C, area b.). The histone deacetylase, *Rpd3 *and the Brahma complex subunit, *Bap55 *fell into this category (Additional file 1A). Hits that scored only for Notch induced signal (NΔecn >*m3-luc*) normalized by the uninduced *E(spl)m3 *promoter (*m3-luc*) represent genes that primarily affect uninduced reporter transcription, such as the repressor complex component *Hairless *and the Brahma complex chromatin remodeling factor *moira *(Figure 2C, area c.).

### Classification of the identified proteins

Classification of modifiers identified in the screen was based upon gene ontologies (GO terms) as reported by Flybase [5]. These classes are shown as a percentage of genes with that GO term and median z-scores of that class (Additional file 3). Certain classes showed particularly significant z-scores. For instance, activators of Notch induced transcription as normalized by the control reporter (*con-luc*) contained 10 chromatin-associated factors, 6.5% of the hits, and 16 transcription factors, representing 10.5%. Both these classes have a median z-score of -2.9, placing these groups in the top 0.2% of the calculated genome-wide distribution (Additional file 3C). Of the identified genes, 90 have predicted and known human orthologs associated with human genetic disorders (Additional file 4) [6].

### Known Notch pathway interactors found by the RNAi screening method

Thirteen genes that have been described to genetically interact with Notch were identified (Figure 2C, boxed gene names). Among these, the core Notch pathway transcription factor *Su(H) *and the repressor *Hairless *further validated the screening method. We also recovered the known negative regulator of Notch signaling, *Suppressor of deltex *[*Su(dx)*], encoding a cytoplasmic protein that functions as an E3 ubiquitin ligase that ubiquitinates membrane-anchored Notch [7], and *prickle *(*pk*), encoding a transcription factor known to play a role in *E(spl)mδ *gene expression [8]. Nine other genes (*heph, al, Sos, toe*, *Vg*, *Lobe, lid, Nipped-A *and *RpL19*) were identified that have been shown to genetically interact with Notch signaling, but whose mechanistic level of integration into the Notch pathway are understood to varying degrees [9-17].

An *in vivo *RNAi screen for Notch activity has recently been published that is based on bristle and wing morphology and as a different approach to this transcriptional based study, the overlap was minimal [18]. Of the 14 genes listed in the previous study that have known genetic interactions with Notch, only tramtrack (ttk) is common to both screens. The direct transcription based method of our study would be expected to be better suited to identify transcription and chromatin factors, as indicated by the strong scores of repressor components and core chromatin components identified (Additional file 3). In contrast, the phenotype-based study was more sensitive to membrane trafficking machinery, making the two studies complimentary.

### Protein interaction network of Notch transcription modifiers

**Figure 3:**
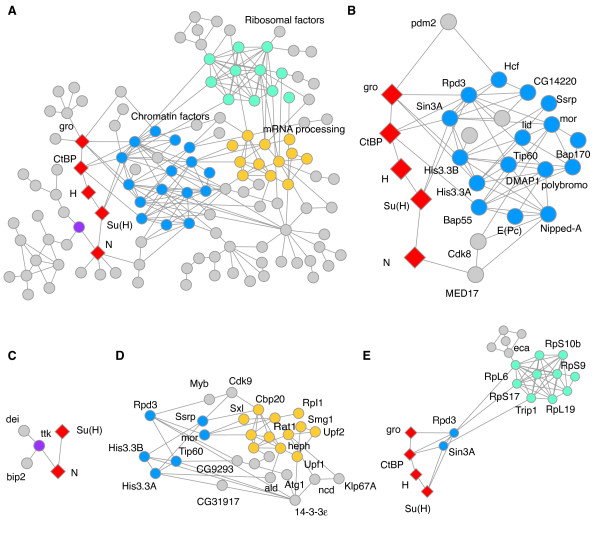
**Protein-protein interaction map of Notch transcription modifiers**. The Notch interaction network was generated by connecting the Notch transcription modifiers identified in the genome-wide study with protein-protein interaction links (e.g. two-hybrid and Co-IP data from the DroID database [19]). This resulting network included 126 genes (nodes) with 237 physical interactions (edges). Genetic interactions were not used for the network and the resulting map was drawn using Cytoscape [51]. **A**. These physical links are shown in relation to components of the activated Notch pathway (N and Su(H)) and the Notch repressor complex (Su(H), H, CtBP and gro), shown in red. **B**. Expanded view of the chromatin factors identified in this study that form the central core of the interaction network (blue). **C**. Ttk is a known downstream target of Notch signaling. The transcriptional and physical interaction data suggests that this factor may have a positive feedback role in Notch induced transcription. **D**. Factors with roles in mRNA processing (yellow). The interaction network suggests that these proteins may be working though the chromatin machinery to modulate Notch transcription. **E**. The interaction network suggests the possibility of a similar chromatin based mechanism for the class of ribosomal proteins known as *Minute*. The network file is included with the supplemental data (Additional file 6) and can be viewed in detail using the open source Cytoscape viewer http://www.cytoscape.org.

An interaction network was generated to map physical interactions between the Notch transcriptional activity modifiers identified in the screen and core components of the Notch signaling pathway (Figure 3). This interaction map was generated by combining physical interaction data (e.g. two-hybrid and Co-IP data) from the DroID [19] and BioGRID [20] databases with the Notch-dependent transcription modifiers identified in this genome-wide study. This map does not include the known genetic interactions identified between the candidate genes and caution should be noted as to the presence of possible false positives in the protein interaction data.

The importance of chromatin in Notch regulation has recently become apparent and this transcription-based screen was suited to uncover this class of regulators. On average, chromatin-modifying genes scored relatively high in the data analysis (Additional file 3). The interaction map reveals a central core of chromatin modifying components that have multiple physical connections to the nuclear elements of the Notch pathway such as Su(H) and H (Figure 3B). Many of these chromatin components are known to interact genetically and physically with the Notch pathway [14,21-23].

The protein interaction network also shows a number of protein classes that have no known mechanistic link to Notch transcriptional regulation. For these classes of molecules (mRNA processing proteins and ribosomal factors, discussed later), the network suggests that they may be affecting Notch signaling through direct interactions with these core chromatin components (Figure 3D and 3E).

### Epistatic analysis of candidate genes

**Figure 4:**
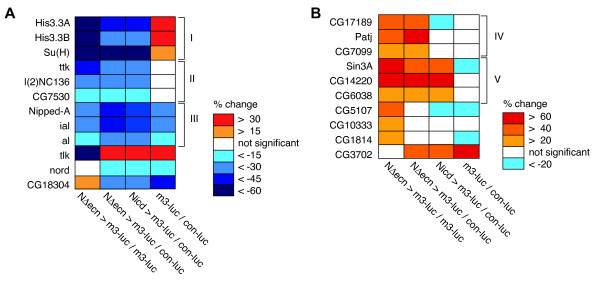
**Analysis of retested genes**. **A**. Retested genes (from selected set, Figure 2C area a) that show significantly reduced signaling when down regulated by RNAi. Signals are shown as (+/-) percent deviation from the control RNAi signal. Three general classes are shown. All three classes down regulate both soluble (Nicd) and membrane bound (NΔecn) Notch-induced signal, yet have different effects on the *E(spl)m3 *promoter in the absence of active Notch. Class I genes have positive, Class II neutral and Class III negative effect on the uninduced signal. **B**. Selected set of retested genes that show significantly enhanced signaling when down regulated by RNAi. Two classes of hits are noted. Class IV is only effective on the membrane bound form of Notch (NΔecn), while class V is effective on both membrane bound and soluble forms (Nicd). All deviations are calculated to be significant by two-tailed t-test with p-values < 0.05 from control RNAi treatment (Additional file 5 for full statistics).

**Figure 5:**
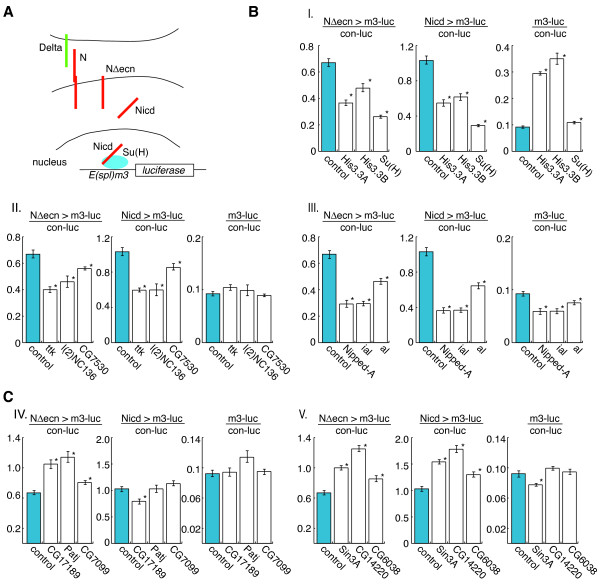
**Modulation of Notch transcription for subset of retested genes**. **A**. The two constitutively active Notch constructs used to determine epistatic relationships in the pathway, NΔecn and Nicd. NΔecn is a truncated form of N missing the extracellular domain that is initially membrane bound. NΔecn undergoes constitutive cleavage to form the soluble Nicd that is transported to the nucleus to activate transcription. Su(H) is the canonical Notch pathway transcription factor that represses transcription in the absence of Nicd and is essential for the Nicd activated transcription of targets such as *E(spl)m3*. **B**. Transcriptional response to RNAi treatment of selected retested genes that promote Notch signaling. The *E(spl)m3 *reporter was induced with either NΔecn or Nicd or left in the uninduced repressed state. All three classes down regulate both soluble (Nicd) and membrane bound (NΔecn) Notch-induced signal, yet have different effects on the *E(spl)m3 *promoter in the absence of active Notch. Class I genes have positive, Class II neutral and Class III negative effect on the repressed signal. **C**. Transcriptional response to RNAi treatment of selected retested genes that repress Notch signaling. Two classes of hits are noted. Class IV is only effective on the membrane bound form of Notch (NΔecn), while class V is effective on both membrane bound and soluble forms (Nicd). Error bars represent the standard error of the mean (SEM). *Significant deviation from control RNAi treatment, calculated by two-tailed t-test with a p-value < 0.05 (Additional file 5 for full statistics).

The subset of candidate Notch modifiers that overlapped between the two normalization methods (28 genes) was retested with redesigned dsRNAs (Additional file 5). Luciferase reporter activity was assessed in cells in which Notch had been activated by either the membrane tethered NΔecn or the downstream intracellular Nicd, aiming to discriminate between factors that regulate Notch processing at the plasma membrane versus factors that affect Notch signaling downstream in the nucleus (Figures 4 and 5). Of the re-designed dsRNA, 79% retested by either normalization method, 67% re-tested the *m3-luc *normalized signal and 64% the *con-luc *normalized signal. Three genes were identified that exclusively promote the activity of the membrane bound Notch and may function to inhibit the intramembrane proteolysis of the receptor. This class includes *Patj *and two genes of unknown function, *CG7099 *and *CG17189 *(Figure 4B, Class IV and Figure 5C, Class IV). The soluble protein Patj has not been shown to modulate Notch activity directly, but is known to associate with the transmembrane protein Crumbs that, in turn, is known to repress Notch activity [24]. Crumbs is a central regulator of epithelial apical-basal polarity in *Drosophila *and has been shown to down regulate γ-secretase activity and the membrane proteolysis of Notch [24]. Our observation in Kc167 cell culture, a non-polarized cell line, suggests that Patj may be modifying Notch signaling not via influencing the localization of the receptor, but instead by acting in the Crumbs-based complex to down regulate membrane proteolysis of Notch. In contrast, RNAi against nuclear factors such as *Su(H), His3.3A *&*B, Nipped-A, ttk *and *Sin3A *(Figures 4 and 5), had similar effects on NΔecn and Nicd induced transcription, indicating interactions with Notch downstream of the proteolytic processing events. These results demonstrate that the screening method identified components of Notch signaling that modulate activities that take place on the plasma membrane as well as nuclear and chromatin-based regulation.

### Factors involved in chromatin modification

The transcription-based screening method using an endogenous *E(spl)m3 *promoter sequence was particularly useful for identifying chromatin components. We identified several chromatin factors previously shown to affect Notch-dependent transcription. A component of the SAGA histone acetyltransferase complex, Nipped-A, was identified. Nipped-A, the *Drosophila *homologue of yeast Tra1 and mammalian TRAP proteins, is a key factor of the SAGA complex. It has been shown previously that reduced Nipped-A dosage enhances the wing notching phenotype of both *mastermind *and *Notch *mutants [14,25]. The RNAi treated cell culture data demonstrates that Nipped-A promotes transcription at the *E(spl)m3 *promoter both in the presence and absence of activated Notch (Figures 4 and 5). This shows that the result of Nipped-A function is independent of whether active Nicd is localized on the target promoter.

We also identified several homologues of components of the Rpd3 histone deacetylase co-repressor complex, including Sin3a, Sds3 (CG14220), a putative ortholog of SAP130 (Sin3A Associated Protein 130, CG11006), and Rpd3 itself (Figures 4, 5 and Additional file 1B). When these factors were targeted by RNAi, there was an increase in Notch-induced reporter transcription, consistent with the role of the Rpd3 complex and histone deacetylation as a transcriptional inhibitor [26]. Conversely, knocking down Sin3a had the opposite effect on the uninduced baseline activity of the *E(spl)m3 *promoter (Figure 5C). Thus, unlike the histone acetylation complex (SAGA), the activity of the deacetylation complex (Sin3A) on the *E(spl)m3 *promoter is dependent on the presence of activated Notch.

The screen identified several components of the chromatin remodeling complex Brahma: Brm Associated Protein 55 (Bap55), Brm Associated Protein 170 (Bap170), polybromo, and moira (mor). A previous *Drosophila *phenotype based screen has found a genetic interaction between the Notch ligand *Delta *and another component of the Brahma complex, *brahma *(*brm*) [21]. Loss of function *brm *alleles were found to enhance *Delta *mutant phenotypes in eye and bristle development [21]. The various Brahma components identified in this study show a complex array of effects on the transcription of the *E(spl)m3 *promoter, some consistent with previously described loss of function *brm *alleles while others opposing. RNAi directed against *Bap55 *and *polybromo *demonstrated a specific reduction in Notch induced transcription (Figure 2C, area b) that is consistent with the previously observed role of *brm *in Notch signaling during *Drosophila *development [21]. Unexpected are the Brahma subunits identified that modulate transcription from the uninduced *E(spl)m3 *promoter: Bap170 and mor. The screen reveals that both of these components specifically mediate transcription from the uninduced *E(spl)m3 *promoter, while Bap170 activates and mor represses (Figure 2C, area c.).

In addition to chromatin modifying complexes, a new interaction between the histone variant H3.3 and Notch signaling is seen. RNAi treatment of either genomic copy of the H3.3 histone variant (*H3.3A *and *H3.3B*) shows a dramatic decrease in Notch activated transcription (Figures 4 and 5). The histone variant H3.3 has been shown to be incorporated into the promoters of actively transcribed genes in a replication independent process to maintain transcription and its influence on Notch targeted transcription remains to be explored [27].

A major question that arises from these data is, how specific can the identified chromatin factors be to regulating Notch transcription? It has recently been noted that chromatin components are more selective in function than was previously thought. Surprisingly, there are now a handful of examples where modulating the expression of a single target gene can rescue the phenotype associated with a null mutation in a chromatin remodeling complex component [28]. By immunoprecipitation and mass-spec analysis, it has recently been shown that the Notch repressor complex contains a host of chromatin modifying components [22]. These identified components include Sin3A, Rpd3, lid, Bap55 and moira, factors that were also uncovered in this screen as modifiers of Notch target transcription. This repressor complex has been shown to be recruited to Notch target promoters by Su(H) and this interaction may provide a mechanism for targeting the activity of these chromatin factors to Notch signaling [22,23]. This is consistent with the observation that the genetic interactions demonstrated between this repressor complex and Notch were not seen when tested against a host of other signaling pathways [22,23]. Control reporter transcription levels in this study indicated that targeting these chromatin genes by dsRNA did not significantly reduce cell viability and growth over the course of the five-day RNAi incubation in culture. The screen data shows that Notch signaling may be particularly sensitive to the levels of these chromatin components in the cell, while the protein interaction network confirms that many of these chromatin factors physically interact with Su(H) and Hairless suggesting a mechanism to explain this observation.

Regulation of histone position and modification are known factors that determine the "context dependent" nature of Notch signaling during development. These factors differentially interpret the signals received from the cell surface by recording an epigenetic history on the target promoter. This transcription-based screen revealed new chromatin factors that can be further studied for their role in Notch-mediated development.

### mRNA processing factors

The genome-wide transcription assay revealed two other classes of proteins not conventionally associated with transcriptional regulation. A number of ribosomal components and proteins associated with mRNA processing were found to regulate transcription of the activated Notch target gene (Figures 2 and 3). What is unexpected about these interactions is their relative specificity, as was for the chromatin components. Again, any RNAi treatments in the genome screen that significantly effected cell viability or general transcription were excluded from the analysis. In addition, all Notch induced target transcription signals were subsequently normalized to either the control signal or the uninduced Notch target promoter. This analysis demonstrated that knocking down these components of the ribosome and splicing machinery did not significantly affect general cell viability and had a relatively specific effect on Notch target transcription.

A number of mRNA splicing and processing components were found to interact with Notch-activated transcription (Figure 2A and 2C). As expected, these proteins demonstrated extensive physical interactions with each other (Figure 3D). Unexpectedly, these mRNA modifying proteins show physical interactions with the core chromatin components identified in this transcription based screen (Figure 3D). The polypyrimidine tract binding proteins Sex lethal (Sxl) and hephaestus (heph) were found to repress and activate Notch promoter activity, respectively, in our cell culture assay. Heph was previously found to interact genetically with Notch signaling during wing development [12]. Other mRNA processing components, such as the non-sense mediated decay factors Upf1, Upf2 and Smg1, were found to modulate Notch activated transcription in the analysis (Figures 2 and 3D). These mRNA components may be interacting indirectly with Notch transcription through their mRNA processing functions - for instance, by specifically controlling the mRNA processing of transcripts for an essential Notch signaling factor such as Su(H). The network suggests a possible alternate mechanism to explain the interaction between the identified mRNA processing factors and Notch transcription, one that is mediated though the chromatin machinery (Figure 3D).

In plants, components of the nuclear cap-binding complex (including Cbp20) functionally interact with microRNA (miRNA) processing components, such as Ars2, giving these proteins dual roles in splicing and miRNA processing [29]. The role of Cbp20 in miRNA processing was also confirmed in *Drosophila *and mammalian systems [30,31]. The nuclear cap-binding complex component Cbp20 was found to mediate Notch transcription in this study (Figure 2) and demonstrates physical interactions with the chromatin remodeling component Ssrp (Figure 3D). The interaction network suggests that the miRNA processing activity of Cbp20 may be targeted to Notch signaling through interactions with the chromatin remodeling machinery.

### Ribosomal factors and the classical *Minute *mutations

A complex of ribosomal proteins was identified that modulated Notch reporter transcription (Figures 2 and 3E). This class of translation factors included the large ribosomal subunit RpL19 that belongs to the *Minute *genetic class. The *Minutes *are a class of ribosomal gene mutations that are homozygous lethal, delay cellular growth when heterozygous and have a rich history of study [32,33]. Of interest, RpL19 has been shown to be a modifier of Notch signaling [15,34,35]. In fact, the *Minute *class of genes was first described in detail as modifiers of Notch signaling in 1929 [36]. Since then, ribosomal components have been widely observed as effectors of Notch [25,37-39]. The Notch transcription reporter measurements compliment these long-standing, yet mechanistically unknown, genetic interactions. One mechanism proposed to explain the relatively specific genetic interactions between *Minute *mutations and Notch, is the possibility of specific translational effects. For instance, the translation of long transcripts such as the one encoding Notch itself may be sensitive to lower levels of specific ribosomal components. In contrast, an alternative hypothesis has been presented that these ribosomal proteins may have post-translational effects on key components of Notch signaling [34]. *Minute *protein mutations are not found in the active site of the ribosome, as the peptide synthesis reaction is catalyzed exclusively by RNA in the core, but rather on the surface of the ribosome. Current structural and biochemical studies have demonstrated post-translational roles for these surface coating ribosomal proteins [40]. This includes the folding of nascent peptide chains either directly on the surface of the ribosome or by the co-recruitment of protein chaperones. The protein-protein interaction map suggests that these types of post-translational interactions may be directed towards the core chromatin components of the Notch network (Figure 5E). Such a direct mechanism could explain the transcriptional effects described in this study, as well as the long-standing genetic observations between Notch and the *Minute *class of mutations.

### Transcription factors that affect Notch-dependent transcription

Analysis of the genes identified in the screen revealed a number of transcription factors that affect Notch-dependent transcription. Among these are cnc and maf-S that are known to form a strong transcriptional activator complex [41]. RNAi targeting of either of these two genes strongly suppressed both the Notch-induced as well as non-induced *E(spl)m3 *reporter activity (Additional file 1A). Also, among the 15 transcription factors that promote Notch activity, we found the DNA binding protein Deaf-1. Cnc, maf-S, and Deaf-1 are reported to interact with the Hox protein Deformed (Dfd) to regulate segmentation, but their roles in other developmental events are not known [42]. Our results provide a possible role of these proteins in *Drosophila *development by promoting Notch signaling.

Another transcription factor that we found to play an agonistic role in Notch signaling is the homeobox containing protein Aristaless (al) (Figure 5B). Al has been tentatively linked to Notch signaling, as it cell autonomously represses the Notch ligand Delta in the pretarsus during leg morphogenesis [10]. It is possible that al is involved in a Notch-mediated lateral inhibition mechanism, where al expressing cells remain undifferentiated by favoring active Notch signaling whereas their neighboring cells are free to express Delta and differentiate. It has also been shown that Notch mutant clones in the developing leg disk show diminished al levels, suggesting that *al *is a Notch target gene. This would be the predicted relationship in a lateral inhibition system, where a Notch/al positive feedback loop would amplify the Notch activity differences between neighboring cells.

Two additional transcription factors that have been previously shown to be involved in leg morphogenesis were found to promote Notch signaling: Bonus (bon), a homologue of the vertebrate TIF1beta transcriptional cofactor [43], and crooked legs (croI), a zinc finger protein [44]. Notch signaling is known to play an important role in *Drosophila *leg development, and the recovery of these two transcription factors as modifiers of Notch-induced *E(spl)m3 *expression suggests that bon and croI may function to modulate Notch target gene output in the developing leg [45].

We also identified the *Drosophila *orthologues of two mammalian proto-oncogenes kayak (c-fos), and c-Myb, as positive regulators of Notch-signaling. Although a direct functional link between these proteins and Notch signaling has not been described, *kayak *has been shown to interact genetically with *Hairless *[46] and *c-Myb *genetically interacts with *bon*, a novel Notch modifier described above [47]. In addition, our data reveals a synergistic relationship between the positive regulator of Ras signaling, 14-3-3ε, and Notch. Once again, the protein interaction network shows extensive contacts between 14-3-3ε and the chromatin machinery, suggesting a mechanism for modulating Notch target transcription through Su(H) mediated chromatin modifications. Interactions between Notch and oncogenic pathways are of particular interest, as the involvement of Notch in cancer biology and stem cell maintenance is becoming increasingly apparent.

An unexpected Notch target transcription modifier identified in the screen is the Notch target gene *Tramtrack *(*ttk*). We found that targeting of *ttk *with dsRNA resulted in reduced Notch activity (Figures 4 and 5). In contrast, *ttk *expression itself has been shown to increase in response to ectopic Notch activity [48]. The RNAi treatment data suggest that ttk may function in a positive feedback mechanism to promote Notch activated transcription and the network analysis suggests that this interaction may be mediated by a direct contact with Notch itself (Figure 3C).

## Conclusions

A complementary, genome-wide RNAi approach has revealed a subset of factors that modulate Notch target transcription that may not have been found by traditional genetic approaches. For instance, pleiotropic effects combined with non-saturating mutagenesis may have obscured the detection of some components in traditional genetic screens. Several novel modifiers were identified in this RNAi transcription-based screen, and these factors will be further investigated for their precise roles in the regulation of Notch signaling during development. In addition, the interaction network of these factors suggests that many may work through contacts with chromatin machinery components that are in turn directed to Notch target promoters by the transcription factor Su(H).

## Methods

### DNA constructs

Constitutively active Notch constructs were made with cDNA encoding either membrane-tethered, *Drosophila *Notch (NΔecn), constitutively activated by the removal of the extracellular domain, or the soluble intracellular domain (Nicd) [2]. These truncated Notch constructs were cloned into the pIZ-V5/His expression vector (Invitrogen) producing non-tagged proteins. The control expression plasmid (*con-luc*) was constructed by cloning firefly luciferase into the same pIZ-V5/His expression vector. The luciferase reporter construct (*m3-luc*) contains a 1.4 kb tandem duplication of *E(spl)m3 *upstream regulatory sequences, cloned into a pGL2-Basic vector (Promega), as described [3].

### Genome-wide RNAi method

A total of 23,560 dsRNAs, made available from the *Drosophila *RNAi Screening Center (DRSC) at Harvard Medical School, were screened by the following method: Kc167 cells were washed three times and resuspended in serum-free Sang's M3 medium (Sigma) at a concentration of 5 × 10^5 ^cells/ml. Using a robotic liquid handler, 10^4 ^cells (20 ml) were uniformly dispensed into the wells of 384-well polypropylene plates containing dsRNA and incubated for 45 min at room temperature. An equal volume of M3 medium containing 10% fetal bovine serum was added and incubated for four days. On day four, the RNAi-treated cells were diluted with 100 μl of medium, mixed and 20 μl were dispensed into the wells of six new 384-well plates, pre-aliquoted with 20 μl of transfection mix. The six plates contained the three different transfection mixes, each in duplicate. Transfection mixes were prepared with Effectene Transfection Reagent (Qiagen), following the manufacturer's guidelines. Luciferase activity was measured 24 h post transfection using the Steady-Glo Luciferase Assay System (Promega).

This method requires only two plasmids to be transfected at one time and gave acceptable signal to noise ratios for high throughput screening in 384-well plate format (Figure 1). Whereas, the conventional renilla dual-glo assay was not robust enough to scale to 384 well format with this Notch reporter system using an endogenous target. In contrast to pathways with soluble ligands, the reporter and constitutively active Notch constructs are required to transfect the same cell to activate transcription. With the renilla dual-glo system, adding the control construct required the co-transfection of three individual plasmids and this reduced the signal to noise ratio to insufficient levels.

### Data analysis

Duplicate measurements for each of the three signals were averaged; Notch specific *E(spl)m3 *promoter in the presence of activated Notch (NΔecn >*m3-luc*), the *E(spl)m3 *promoter alone (*m3-luc*) and the unrelated viral promoter OplE2 (*con-luc*). The NΔecn >*m3-luc *signal was normalized two different ways, by either the *m3-luc *or *con-luc *signals. The z-scores of the log_2 _ratios were calculated by using the standard deviation and mean of the measurements that corresponded to the 96-wells of the original dsRNA stock plates [49]. To remove ratios that contained data that did not sufficiently replicate in the original duplicate measurements, a distribution was calculated for the individual errors (estimated from the duplicate data sets) and ratios with an associated error z-score above 3 were removed from further analysis (0.9% of the total data).

To remove data associated with dsRNA that greatly reduced general transcription or cell viability, a distribution of the signals from the control promoter (*con-luc*) was calculated, and data with z-scores below -2 were removed (4% of the total wells). All calculations were done by in house software written in JAVA.

Hits were chosen as those log_2 _ratios with a z-score above 2 or below -2 for NΔecn >*m3-luc *normalized by the viral promoter OplE2 (*con-luc*). For the data set normalized by the *E(spl)m3 *promoter alone (*m3-luc*), a z-score above 1.8 or below -1.8 was used. The *m3-luc *normalized distribution had more defined outliers indicating a better data set. As a consequence, *m3-luc *normalized data distribution had higher kurtosis as seen by a slightly sharper peak in Figure 2. This does not change the rank order or relative differences in the hits of that data set, but to make the cut-offs more equivalent between the two normalization methods, the different cut-off values were used.

### RNAi retest procedure

Genes were chosen for retesting that were selected as positive by both normalization methods (Figure 2c, area a.). This second set of 28 dsRNAs were independently redesigned by the method of Arziman et al. with no predicted off targets and are listed in Additional file 5[50].

DNA templates for T7 reactions were generated by PCR from Kc167 cell genomic DNA and dsRNA was produced using the MEGAscript RNAi kit (Ambion). Per well, 25 ml of Kc167 cells at a concentration of 8 × 10^5 ^cells/ml were incubated with 1.25 μg of dsRNA (0.5 mg/ml stock) for 1 h in serum-free M3 medium. M3 medium with 10% FBS (75 μl) was then added and incubated for 4 days. On the fourth day, 125 μl of medium was added, and treated cells were split into 4 wells with 50 μl per well, each containing 50 μl of the following transfection mixes, prepared as above: a. *con-luc*, b. *m3-luc*, c. *m3-luc *& pIZ-NΔecn, d. *m3-luc *& pIZ-Nicd. Luciferase levels were measured after 25 h, as above. Retests were done in quadruplicate for each dsRNA, and the results are given in Additional file 5 for the 22 positive retests that have p-values < 0.05 (compared with control dsRNA treated cells).

### Notch interaction network construction

The Notch interaction network was generated by combining physical interaction data (e.g. two-hybrid data) from the DroID database [19] (that contained interactions from the BioGRID database [20]) with Notch transcription modifiers identified in the genome-wide study. Genetic interactions were not used for the network map. The resulting network was drawn using Cytoscape [51] and the data can be found in additional file 6.  The network file can be viewed in detail using the open source Cytoscape viewer http://www.cytoscape.org.

### Orthology prediction

All orthology predictions for candidate genes were made using InParanoid [52].

## Authors' contributions

PM, RL and BD designed and performed experiments, analyzed data and wrote the manuscript. CF performed experiments and analyzed data. All authors read and approved the final manuscript.

## Supplementary Material

Additional file 1 **A. Excel table of screening data for Notch induced transcription normalized by the unrelated control promoter (NΔecn > m3-luc/con-luc)**. Hits listed from initial screen with potential agonists of Notch induced transcription listed in **A**. and potential antagonists listed in **B**. Click here for file

Additional file 2 **Excel table of screening data for Notch induced transcription normalized by the uninduced *E(spl)m3 *promoter (NΔecn > m3-luc/m3-luc)**. Hits listed from initial screen with potential agonists of Notch induced transcription or antagonists of uninduced *E(spl)m3 *transcription listed in **A**. and vice versa in **B**. Click here for file

Additional file 3 **Figure representing the distribution of Notch modifiers by gene ontology classes**. Pie chart distributions for percentage of genes represented in major gene ontology classes and corresponding box plots of median z-scores for the various classes. Box plots are graphed as in Figure 1a. **A**. Distribution of genes that enhance the Notch induced signal as normalized by the uninduced *E(spl)m3 *promoter. **B**. Distribution of genes that suppress the Notch induced signal as normalized by the uninduced *E(spl)m3 *promoter. **C**. Distribution of genes that enhance the Notch induced signal as normalized by the unrelated control promoter (*con-luc*). **D**. Distribution of genes that suppress the Notch induced signal as normalized by *con-luc*. Click here for file

Additional file 4 **Excel table of orthology predictions**. Human orthologs of the *Drosophila *genes identified in the screen were predicted using InParanoid [52]. Human diseases associated with the predicted genetic counterparts are also listed. Click here for file

Additional file 5 **Excel table of re-test data for the redesigned dsRNA**. Genes were chosen for retesting that were selected as positive by both normalization methods (Figure 2c, area a.). This second set of 28 dsRNAs were independently redesigned by the method of Arziman et al. with no predicted off targets and are listed in Additional file 5[50]. Retests were done in quadruplicate for each dsRNA, and the results are given for the 22 positive retests that have p-values < 0.05 (compared with control dsRNA treated cells). Click here for file

Additional file 6 **Notch interaction network file**. A network file that can be viewed in detail using the open source Cytoscape viewer http://www.cytoscape.org[51]. The Notch interaction network was generated by using Notch transcription modifiers identified in the genome-wide study as nodes and physical interactions (e.g. two-hybrid data) for edges. Genetic interactions were not used for the network map. Click here for file
