# Supplementary material for: Modifiers of notch transcriptional activity identified by genome-wide RNAi
Source: BMC Dev Biol. 2010 Oct 19;10:107. doi: 10.1186/1471-213X-10-107 (PMC2976970; doi:10.1186/1471-213X-10-107)
Supplement: Additional file 3 — Figure representing the distribution of Notch modifiers by gene ontology classes. Pie chart distributions for percentage of genes represented in major gene ontology classes and corresponding box plots of median z-scores for the various classes. Box plots are graphed as in Figure 1a. A. Distribution of genes that enhance the Notch induced signal as normalized by the uninduced E(spl)m3 promoter. B. Distribution of genes that suppress the Notch induced signal as normalized by the uninduced E(spl)m3 promoter. C. Distribution of genes that enhance the Notch induced signal as normalized by the unrelated control promoter (con-luc). D. Distribution of genes that suppress the Notch induced signal as normalized by con-luc. [file 1471-213X-10-107-S3.PDF]

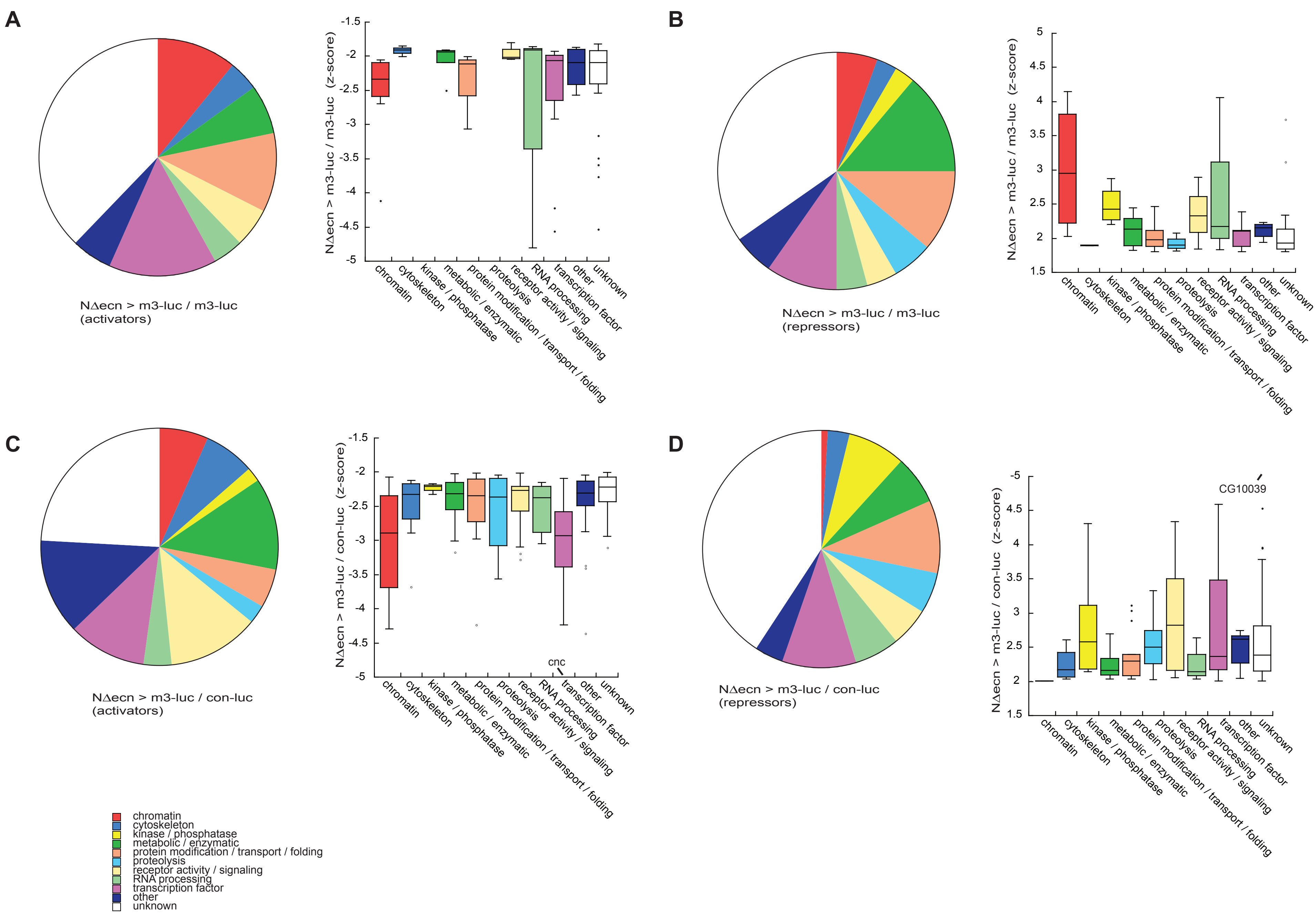

A
NΔecn > m3-luc / m3-luc
(activators)
NΔecn > m3-luc / m3-luc (z-score)
-1.5
-2
-2.5
-3
-3.5
-4
-4.5
-5
chromatin
cytoskeleton
kinase / phosphatase
metabolic / enzymatic
protein modification / transport / folding
proteolysis
receptor activity / signaling
RNA processing
transcription factor
other
unknown
B
NΔecn > m3-luc / m3-luc
(repressors)
NΔecn > m3-luc / m3-luc (z-score)
5
4.5
4
3.5
3
2.5
2
1.5
C
NΔecn > m3-luc / con-luc
(activators)
NΔecn > m3-luc / con-luc (z-score)
cnc
D
NΔecn > m3-luc / con-luc
(repressors)
NΔecn > m3-luc / con-luc (z-score)
-5
4.5
4
3.5
3
2.5
2
1.5
CG10039
chromatin
cytoskeleton
kinase / phosphatase
metabolic / enzymatic
protein modification / transport / folding
proteolysis
receptor activity / signaling
RNA processing
transcription factor
other
unknown
